# The Great Northern Central Hospital

**Published:** 1893-05-27

**Authors:** 


### FESTIVAL DINNERS AND MEETINGS.

### THE GREAT NORTHERN CENTRAL HOSPITAL.

It was a remarkable gathering at the Hotel Metropole on Thursday, 18th inst, when H.R.H. the Duke of York accepted the Presidency of this important hospital. Those who dined were for the most part resident in the district served by the hospital. The dinner was therefore an important occasion for the guests as also for the hospital, and this was shown by the reception given to the Duke of York. There was a diffidence about the applause with which the toast of his health was received, which might have been put down by the inexperienced to a want of enthusiasm. That such a view was wholly opposed to the facts was proved by the heartiness of the cheers, the whole assembly rising, when the Chairman of the hospital, Mr. Murdoch, after the Duke's acceptance of the office of President, called for three cheers for His Royal Highness, with one cheer more for the Princess May. The Great Northern Central dinner was the first festival of the kind at which the Duke of York had appeared since his engagement, and no more fitting occasion could have been found for his first appearance, seeing that this hospital is unique amongst metropolitan hospitals. It is unique because it is the first large hospital which has been removed from the relatively small area in which most of the great metropolitan hospitals are situated to the centre of the district from which its patients are received. When completed it will be unique amongst the hospitals of the world, because it will be the first hospital ever built which contains circular and rectangular wards of exactly similar proportions and surroundings where it will be possible to test the relative advantages of these two systems of construction for crowded city sites. We congratulate his Royal Highness and all connected with the hospital upon the splendid success of this dinner. The toast list was short but comprehensive; the speeches were on the whole excellent; and the monetary results remarkable, for no less than £5,425 was received, including £50 from the Queen and £25 from the Duke of York. This dinner may, therefore, be regarded as the most successful of the season, and we hope it may indicate the completeness of the happiness and the continuous success which are in store for H.R.H. the Duke of York and the Princess May.

The Duke of York, who was supported amongst many other influential personages by the Earl of Meath, Lord Colville of Culross, Lord Kinnaird, and Sir W. S. Savory, F.R.S., proposed the toast of the evening, "Success and Prosperity to the Great Northern Hospital." Perhaps, he said, they would allow him to give a short history of the hospital since it was founded in 1856. The first building was situated at King's Cross, and was for out-patients only. Two years afterwards it was provided with ten beds for in-patients. In 1862 the site was taken by the Metropolitan Railway, and temporary arrangements had to be made until 1864, when a site in the Caledonian Road was obtained, and the work was carried on there under great difficulties for over 20 years. The rapid increase of the population of North London, and the development of the numerous railway systems in the district, which added to the number of accident cases, made it imperative to provide further accommodation. The present site in the Holloway Road was purchased, and plans for a building to contain 150 beds were designed. But, as the necessary funds were not forthcoming, it was decided to erect only half, containing 72 beds; and this building was opened by his parents in 1888. The increased accommodation, however, failed to meet the growing wants of the district; and therefore it was determined to finish the building at a cost of £30,000. Of this sum only £10,000 had been collected, out of which £8,000 had already been paid to the contractors, so that there still remained a debt of £20,000. With reference to the want of accommodation, he might mention that last year 23,600 new patients applied for treatment. and the attendences at the out-patient department numbered about 53,000. These numbers spoke for themselves. (Cheers.) In giving this brief history of the hospital it would not be out of place if he alluded to a few of those who had worked so hard for its welfare—namely, Dr. Cholmondeley, the Hon. R. Capel, and the chairman, Mr. Murdoch. (Cheers.) He must also not omit to mention the great and valuable assistance rendered by the ladies' committee, through whose exertions funds were collected to build the present out-patient department, which he believed to be second to none in this great metropolis. (Cheers.) In conclusion it needed no words of his to recommend this great charity, not only to the large and populous district of North London, but also to those he saw around him that evening. It seemed to him that no charities should appeal more to one's sympathies than these great institutions, which did so much to alleviate the pain and suffering of the sick and poor, and that there were none which had greater claims on their support. (Cheers.)

The toast was coupled with the names of Mr. Murdoch and Mr. Burdett-Coutts (the Treasurer), who briefly replied, and the former of whom announced, amid loud cheers, that the Duke of York had consented to become President of the institution.

Sir William Savory spoke to the toast of "The Medical Staff," and in doing so recognised the importance of hospitals to the furtherance of medicine and surgery.

